# Timing Is Critical for an Effective Anti-Metastatic Immunotherapy: The Decisive Role of IFNγ/STAT1-Mediated Activation of Autophagy

**DOI:** 10.1371/journal.pone.0024705

**Published:** 2011-09-13

**Authors:** Jun Yan, Zi-Yan Wang, Hong-Zhen Yang, Han-Zhi Liu, Su Mi, Xiao-Xi Lv, Xiao-Ming Fu, Hui-Min Yan, Xiao-Wei Zhang, Qi-Min Zhan, Zhuo-Wei Hu

**Affiliations:** 1 Molecular Immunology and Pharmacology Laboratory, State Key Laboratory of Bioactive Substances and Functions of Natural Medicines, Institute of Materia Medica, Chinese Academy of Medical Sciences and Peking Union Medical College, Beijing, China; 2 State Key Laboratory of Molecular Oncology, Cancer Institute and Cancer Hospital, Chinese Academy of Medical Sciences and Peking Union Medical College, Beijing, China; University of Palermo, Italy

## Abstract

**Background:**

Immunotherapy is often recommended as an adjuvant treatment to reduce the chance of cancer recurrence or metastasis. Interestingly, timing is very important for a successful immunotherapy against metastasis, although the precise mechanism is still unknown.

**Methods and Findings:**

Using a mouse model of melanoma metastasis induced by intravenous injection of B16-F10 cells, we investigated the mechanism responsible for the diverse efficacy of the prophylactic or therapeutic TLR4 and TLR9 agonist complex against metastasis. We found that the activation of TLR4 and TLR9 prevented, but did not reverse, metastasis because the potency of this combination was neither sufficient to overcome the tumor cell-educated immune tolerance nor to induce efficacious autophagy in tumor cells. The prophylactic application of the complex promoted antimetastatic immunity, leading to the autophagy-associated death of melanoma cells via IFNγ/STAT1 activation and attenuated tumor metastasis. IFNγ neutralization reversed the prophylactic benefit induced by the complex by suppressing STAT1 activation and attenuating autophagy in mice. However, the therapeutic application of the complex did not suppress metastasis because the complex could not reverse tumor cell-induced STAT3 activation and neither activate IFNγ/STAT1 signaling and autophagy. Suppressing STAT3 activation with the JAK/STAT antagonist AG490 restored the antimetastatic effect of the TLR4/9 agonist complex. Activation of autophagy after tumor inoculation by using rapamycin, with or without the TLR4/9 agonist complex, could suppress metastasis.

**Conclusion and Significance:**

Our studies suggest that activation of IFNγ/STAT1 signaling and induction of autophagy are critical for an efficacious anti-metastatic immunotherapy and that autophagy activators may overcome the timing barrier for immunotherapy against metastasis.

## Introduction

The main cause of cancer mortality is disseminated disease, rather than the primary tumor [1]. Conventional treatments, such as surgery, radiotherapy and chemotherapy, have little effect on metastasis and recurrence, especially if a large proportion of the tumor has already metastasized at the time of diagnosis. Thus, metastasis remains the most formidable challenge in cancer therapy. Metastasis is determined by the interaction between the tumor cells and the host tissue microenvironment [2]. Immunotherapy is particularly well suited to eliminate residual tumor cells, especially quiescent and cancer stem cells because immunotherapy manipulates the microenvironment to induce cancer immunity, thus eradicating metastatic tumor cells [3].

Numerous anticancer immunotherapeutic strategies have been developed, including active immunization (i.e., cancer vaccines and adjuvants), passive immunization (i.e., adoptive cell immunotherapy), and antibodies and small molecular inhibitors that modulate the tumor microenvironment [4]. However, the clinical results of immune-based strategies for treating human cancer have not met expectations. This limited success is largely attributed to the immune tolerance observed in cancer patients [5]. Indeed, during tumor progression, increased immunosuppressive factors and immune evasion protect the host from the induction of an efficacious anti-cancer response by immunotherapy [6]. Furthermore, the timing for immunotherapy is another key factor for determining the outcome of the therapy; however, the mechanism underlying this remains unclear.

Toll-like receptors (TLRs) are a family of conserved pattern-recognition receptors (PRRs) that mediate the inflammatory response by detecting conserved motifs of pathogen- or damage-associated molecular patterns (PAMPs or DAMPs) [7]. Both developed and emerging TLR agonists for cancer treatment act as stand-alone therapies or in combination with various agents [8], [9]. However, anticancer responses are often not achieved under physiological conditions [10], and numerous TLR-based immunotherapy strategies for cancer treatment eventually fail [11]. The clinical impact of these studies is highlighted by the recent failure of the Stage III clinical trial of CpG 7909 in non-small cell lung cancer [12]. Both the TLR4 agonist *Escherichia coli* lipopolysaccharide (EC-LPS) and the TLR9 agonist CpG oligodeoxynucleotide (CpG ODN) are immunostimulants and can induce a potent Th1-type immune response *in vivo*. Moreover, TLR4 acts in synergy with TLR9 in the induction of IL-12p70 in mouse dendrite cells (DCs) [13], [14]. We therefore designed an immunotherapeutic regimen consisting of EC-LPS plus CpG ODN to assess the effect of this potent immunotherapy regimen in a metastatic mouse model of B16 melanoma cells. Despite an optimal synergistic combination of EC-LPS plus CpG ODN with a similar dose and frequency, only prophylactic (versus therapeutic) administration of this complex attenuated metastasis, indicating that effective antimetastatic immunotherapy depends critically on administration timing. We further investigated what mechanism(s) was responsible for the different efficacy resulting from the timing of the complex's delivery. Our study indicated that perturbation of signal transducers and activators of transcription 1/3 (STAT1/3) and autophagy induction accounted for the complex's distinctive efficacy against metastasis. Our study may provide guidance in designing rational immunotherapeutic strategies for patients with advanced cancers.

## Materials and Methods

### Reagents

*E. Coli* 0111: B4 LPS (Ultra-Pure) was purchased from InvivoGen. CpG ODN 1826 (5′- TCC ATG ACG TTC CTG ACG TT-3′, phosphorothioate-modified) and CpG 1826 control (5′- TCC ATG AGC TTC CTG AGC TT-3′, phosphorothioate-modified) came from Beijing SBS Corporation. FITC-, PE- or PE-Cy5-conjugated anti-CD3, CD4, CD8, CD25, F4/80, CD206, IgG2b and IgG2a mAb, IFNγ, IL-12p70, IL-4, IL-10 and TGF-β1 ELISA kits were purchased from eBioscience (San Diego, CA). Anti-STAT1, p-STAT3 (Ser727), STAT3, suppressor of cytokine signaling 3 (SOCS3), proliferating cell nuclear antigen (PCNA), phosphoinositide 3-kinases 85α (PI3K85α), PI3K110α and Actin antibody were obtained from Santa Cruz Biotechnology, Inc. (Santa Cruz, CA). Anti-p-STAT1 (Tyr701), SOCS1, cleaved caspase-3, beclin 1, histone H3, mammalian target of rapamycin (mTOR), p-mTOR, immunity-related GTPase family M member 1 (IRGM1), glycogen synthase kinase 3 (GSK3), p-GSK3, AKT and p-AKT antibodies were purchased from Cell-Signaling Technology Inc. (Danvers, MA). Anti-LC3B antibody was from Abcam Inc. (Cambridge, UK). Anti-IFNγ antibody (clone XMG1.2) and isotype-matched IgG1 were from BD Biosciences Pharmingen (San Diego, CA). Rapamycin and anti-P62 antibody were from Sigma-Aldrich, Inc. (St. Louis, MO).

### Cell culture

The mouse melanoma cell line B16-F10 (B16 cells, CRL-6475) was cultured in RPMI 1640 (Invitrogen Corporation, Carlsbad, CA) supplemented with 2 g/L Na_2_CO_3_, 100 units/ml penicillin, 50 µg/ml streptomycin, and 10% FBS at 37°C in 5% CO_2_.

### Animal studies

Female C57BL/6 mice were purchased from Vital River Lab Animal Technology, Co. Ltd (Beijing, China) and maintained under standard conditions in an animal facility at the Institute of Materia Medica. Animal care and experimentation were conducted in accordance with guidelines of the Institutional Committee for the Ethics of Animal Care and Treatment in Biomedical Research of Chinese Academy of Medical Sciences and Peking Union Medical College (Permit NO. 002802). All mice used in these studies were between 5 and 6 weeks of age. The tail veins of the mice were injected with 5×10^5^ B16-F10 cells that were resuspended in 200 µl PBS, as previously described [15]. The mice were euthanized with excessive anesthesia at 14 days, and whole lungs were isolated to calibrate the lung weight index (wet lung weight (mg) per body weight (g)). An anatomical microscopic metastasis quantitation was performed by counting the metastasis nodes with different diameters on the surface of all lobes of lungs for each mouse by two observers in a blinded fashion. The lungs were then fixed with 4% paraformaldehyde to prepare for histology analysis.

To determine the impact of immunotherapy on tumor cell metastasis, mice were injected i.p. with TLR4 agonist EC-LPS (12.5 µg/kg) plus TLR9 agonist CpG ODN (0.25 mg/kg) or PBS plus CpG control (0.25 mg/kg) every three days over the seven days before (Days 7, 4, 1) or after (Days 1, 4, 7) tumor cell inoculation [16]. Sham and B16 bearing mice received PBS vehicle alone. To determine the role of STAT1 activation in the protective effect of prophylactic treatment, IFNγ-neutralizing or isotype-matched antibody (100 µg/mouse) was injected intravenously with TLR4/9 agonist complex before tumor inoculation. Alternatively, human recombinant IFNγ (1×10^6^ U/kg) alone was applied to the mice once a day before tumor cell inoculation [17]. To determine the role of autophagy in metastasis, rapamycin (i.p., 10 mg/kg/day) was applied with or without the TLR4/TLR9 agonist complex after tumor cell inoculation [18]. To determine the role of STAT3, the mice were treated with the TLR4/TLR9 complex with or without the JAK2/STAT3 antagonist AG490 (i.p., 30 mg/kg/day) after tumor cell inoculation [19].

### Western blotting

To analyze protein expression, the right lung lobes were lysed in RIPA buffer (50 mM Tris-HCl, pH 7.4, 1% NP-40, 0.25% Na-deoxycholate, 150 mM NaCl, 1 mM EDTA) supplemented with 50 mM NaF, 20 mM β-glycerophosphate and a complete protease inhibitor cocktail (Roche). Cytoplasmic and nuclear fractions were prepared as described previously[20]. Protein concentrations were determined by bicinchoninic acid reagent. Proteins were separated by sodium dodecyl sulfate polyacrylamide gel electropheresis (SDS-PAGE) (12%, 10%, or 8% acrylamide) and transferred to polyvinylidene fluoride (PVDF) membranes. Membranes were incubated overnight at 4°C with primary antibodies. After washing, the blots were incubated with the appropriate HRP-conjugated secondary antibody and processed to detect electrochemiluminescence signals (Amersham Biosciences). The signal intensity was determined with the Gel-pro® Analyzer (Gel-Pro Plus Version 6.0, Media Cybernetics).

### Hematoxylin and eosin (H&E) and immunofluorescence (IF) staining

The left lower lobe of the lung was isolated, fixed, paraffin embedded and coronally sliced into 4-µm thicknesses. The tissue sections were stained with H&E. Protocols for immunofluorescence staining for LC3II and LAMP1 have been described previously [21]. The apoptosis of lung tissues was detected with terminal deoxynucleotidyl transferase (TdT) nick-end labeling (TUNEL) using the DeadEndTM Fluorometric TUNEL System (Promega, WI). The tissues were observed with a Leica SP2 confocal microscope (Leica Microsystems, PA) equipped with the appropriate filter system. The images were analyzed with Leica confocal software. The autophagosomes were evaluated using the coexpression of LC3 and LAMP 1. Autophagy-associated cell death was determined with both LC3 and TUNEL [21].

### Electron microscopy (EM)

The left lower lung lobe was cut into 1-mm^3^ slices and fixed in 2.5% glutaraldehyde in cacodylate buffer (0.1 M, pH 7.2) and in 1% osmium tetroxide (OsO_4_) in cacodylate buffer. After fixation, the slices were dehydrated and embedded in Spurr's resin. For light microscopy, semithin sections (2 mm) were stained with toluidine blue. Ultrathin sections (70 nm) were cut, and structural analysis was performed using an Olympus CM-12 Transmission Electron Microscope operated at 80 KV. To quantify autophagic structures, digital images were acquired. The number of autophagosomes and autolysosomes in the cell cytoplasm was determined per microscope area, and four separate thin sections were analyzed for each mouse.

### Flow cytometry

The lungs were perfused with PBS through the right ventricle, and then whole lungs were harvested and dissected into approximately 1-mm pieces. Single-cell suspensions were prepared with 2 ml of dispase containing collagenase (2 µg/ml) and DNase (50 µg/ml) for 30 min [15]. After RBC lysis, the digested lungs were resuspended in PBS and sequentially filtered through 70-µm filters, and each single-cell suspension was divided into four parts to analyze the number of CTL, Th, M1, M2 and Treg cells in the lung. The cells were incubated with saturated concentrations of FITC-, PE- and/or PE-cy5-conjugated mAb against CD3, CD4, CD8, CD25, Foxp3, CD11b, CD11c, MHC I, MHC II, F4/80 or CD206. Isotype-matched mAbs were used in the control samples. CD4^+^ and CD8^+^ cells were gated from CD3^+^ cells. CD4^+^CD25^+^ Tregs were gated from Foxp3^+^ cells. M1 and M2 cells were gated from CD11b^+^ cells. Data were analyzed using CellQuest software (Becton Dickinson, San Jose, CA).

### ELISA for cytokines in lung tissue

The right lung lobes were lysed in PBS supplemented with complete protease inhibitor cocktail. Lung tissue homogenate was diluted with lysis buffer to a final protein concentration of 500 µg per ml. The expression of IFNγ, IL-12p70, IL-4, IL-10 and TGF-β1 in lung tissue homogenates was detected using ELISA kits according to the manufacturer's instructions.

### Statistical analysis

Differences between groups were assessed by ANOVA. Survival curves were compared by the log-rank test. The results were presented as the mean ± standard error (S.E.). P values <0.05 were considered statistically significant.

## Results

### Timing determines the efficacy of the TLR4/9 agonist complex against metastasis

**Figure 1:**
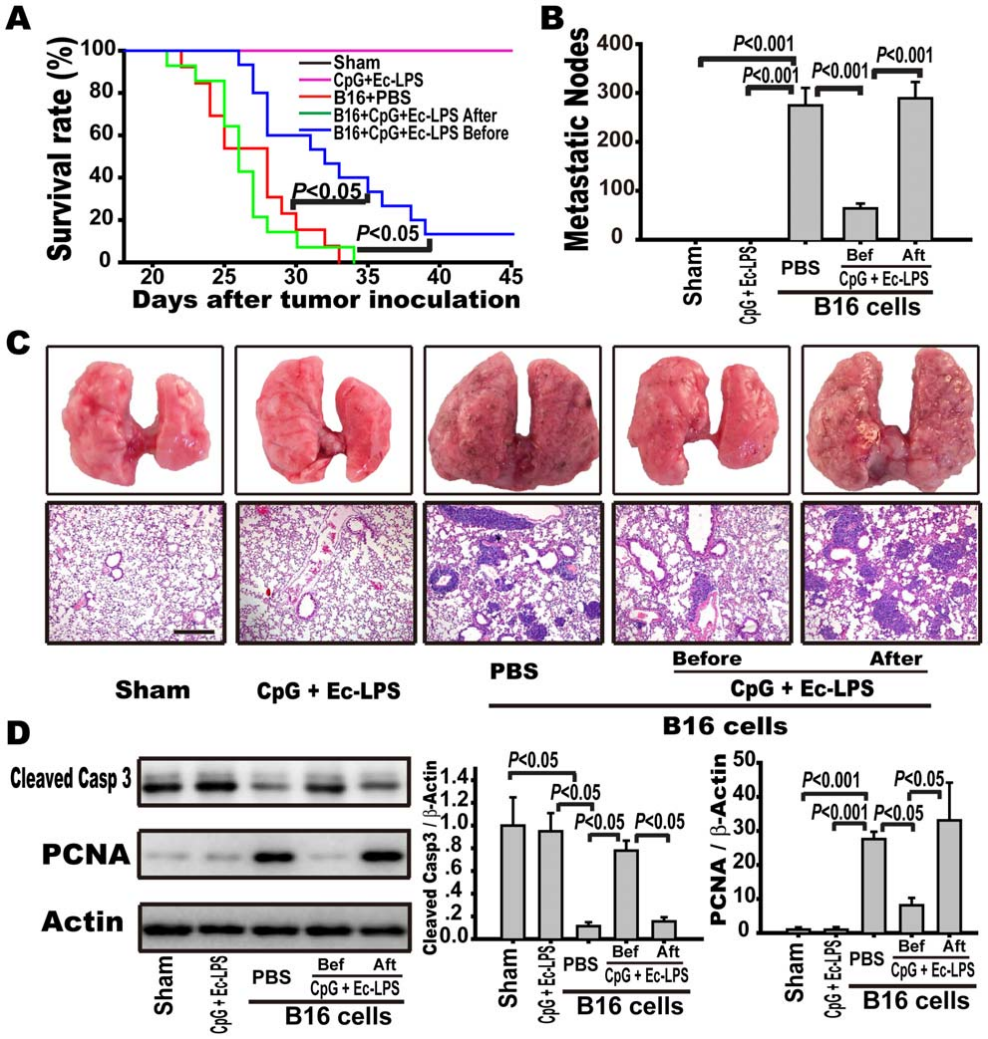
Prophylactic, but not therapeutic, administration of the complex attenuates pulmonary metastasis of B16 melanoma cells. C57BL/6 mice were injected i.v. with B16-F10 melanoma cells (5×10^5^/mouse) and administered with the TLR4 agonist EC-LPS (12.5 µg/kg) plus the TLR9 agonist CpG ODN (0.25 mg/kg) i.p. every 3^rd^ day for 3 doses before (prophylactic group) or after (therapeutic group) tumor cell inoculation. Control animals were treated with PBS or EC-LPS plus CpG ODN with an identical dosage and frequency as indicated in prophylactic group. The mice were humanely sacrificed, and their lungs were excised 14 days after tumor cell inoculation. Externally visible melanoma nodules on the lung surface were counted using stereo microscopy. (A) Kaplan-Meier graph representing the cumulative survival of mice in the indicated treatment groups. The data were analyzed using Kaplan-Meier survival analysis (n = 15 per group). (B) The metastatic nodules were counted and data presented as the mean ± S.E. (n = 15). (C) Data are representative lung samples (upper panel) and representative H&E staining of lung sections (below panel) (magnification: 100×). (D) The expression of cleaved caspase-3 and PCNA was detected by western blot (left panel) and corresponding quantification (right panel) in lung tissues 14 days after inoculation. Data are presented as the mean ± S.E. (n = 5 mice per group).

To investigate the optimal timing for initiating anticancer immunotherapy with the TLR4 agonist EC-LPS plus the TLR9 agonist CpG, mice were injected i.v. with B16-F10 melanoma cells, and the TLR4/TLR9 agonist complex was injected i.p. either before (prophylactic) or after (therapeutic) tumor cell inoculation every three days for three doses. Control mice were treated with PBS or the TLR4/TLR9 agonist complex without B16 cell inoculation. The PBS-treated mice inoculated with B16-F10 cells formed a great number of macroscopic pulmonary metastases two weeks after tumor cell inoculation. The first animal deaths occurred on the 23rd day, and all animals had died by the 34th day after tumor cell inoculation (Fig. 1A, 1B, 1C). Prophylactic administration of the TLR4/TLR9 agonist complex increased the animals' survival rate (40% survival on the 34th day), prolonged the survival time (13% of mice remained alive on the 45th day), and decreased the number of metastatic nodules (64±5 vs. 257±18 nodules/lung, *p*<0.001), compared with the PBS treatment (Fig. 1A, 1B, 1C). However, therapeutic administration of the complex neither suppressed metastasis (280±17 nodules/lung) nor enhanced animal survival (all died on the 33rd day). Thus, prophylactic, but not therapeutic, administration of the TLR4/9 agonist complex attenuated the pulmonary metastasis of B16 melanoma cells.

Many therapies suppress tumor progression by inducing programmed cell death and/or by inhibiting tumor cell proliferation [22]. We thus examined the markers of apoptosis and proliferation in the lung tissue. Two weeks after the final injection of the TLR4/9 agonist complex, the expression of activated caspase-3 and PCNA in the lung tissue of the mice treated with the immune complex was similar to that in the mice treated with PBS in the absence of tumor cell inoculation (Fig. 1D). Prophylactic administration with the TLR4/9 agonist complex induced an increase in the expression of activated caspase-3 (0.78±0.09 vs. 0.11±0.03, *p*<0.05) and a decrease in PCNA expression (8.2±2.1 vs. 27.6±2.0, *p*<0.05), compared to PBS administration in the lung tissues. However, therapeutic application of the TLR4/TLR9 agonist complex neither induced tumor apoptosis (0.15±0.04) nor attenuated tumor cell proliferation (33.1±11) (Fig. 1D). In fact, the therapeutic application of the TLR4/TLR9 agonist complex suppressed caspase-3 activity (0.47±0.16 vs. 1.64±0.33, *p*<0.01) compared to the mice treated with PBS in the early stage of metastasis (Supplementary Fig. S1A). Therefore, two different timing regimens of the TLR4/9 agonist complex had different efficacies against metastasis due to their different capacities for regulating apoptosis and proliferation.

### Prophylactic or therapeutic application of the TLR4/TLR9 agonist complex differentially regulates the inflammatory milieu in the lung of B16 bearing mice

**Figure 2:**
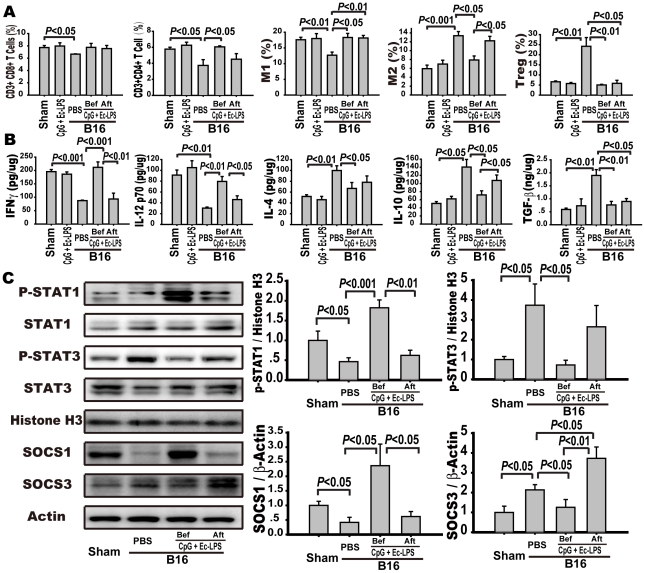
Prophylactic or therapeutic application of the complex differently regulates the inflammatory milieu and STAT1 activation. Mice were treated as indicated in the legend of Figure 1 and sacrificed 14 days after B16 melanoma cell inoculation. Lung single-cell suspensions were prepared as indicated in the Methods. (A) CD3^+^CD8^+^ T cells, CD3^+^CD4^+^ T cells, Foxp3^+^CD4^+^CD25^+^ Treg cells, CD11b^+^F4/80^+^CD206^−^ M1 and CD11b^+^F4/80^+^CD206^+^ M2 macrophages were determined by flow cytometry. Data are the mean ± S.E. (n = 5). (B) The levels of antitumor cytokines IFNγ and IL-12p70 and suppressive factors IL-4, IL-10, TGF-β were detected in lung homogenates from mice using ELISA kits. Data are the mean ± S.E. (n = 5). (C) The expression of STAT1 and STAT3 singling molecules in the lung tissue. The lungs were excised, and the cytoplasmic and nuclear fractions were extracted as described in the Methods. The expression of p-STAT1, STAT1, p-STAT3, STAT3, and histone H3 in nucleic extracts and SOCS1, SOCS3, and β-actin in the cytoplasm were detected with Western blotting. Left panel is representative western blots and right panels are summary results. Data are presented as the mean ± S.E. of five mice per group.

To determine the influence of the complex on the immune system in control animals, mice were injected with PBS or the TLR4/9 agonist complex, and immune responses in lung tissue were examined at 2 weeks after final injection of the complex. We found that the lung-infiltrating immune cells and the expression of cytokines in the mice treated with the complex were similar to those in the mice treated with PBS in the absence of tumor cell inoculation (Fig. 2A, B). We then examined the infiltration of immune cells and the expression of cytokines in the lung tissues after tumor cell inoculation. An immunosuppressive microenvironment was formed in the lung tissues of the PBS-treated B16-bearing mice, with suppressed infiltration or secretion of CD3^+^CD8^+^ T cells, CD3^+^CD4^+^T cells, M1 cells, IFNγ, and IL-12p70 and increased infiltration or secretion of M2 cells, Treg cells, IL-4, IL-10, and TGF-β (Fig. 2A, 2B). Prophylactic intervention induced antitumor immunity in the lung tissues, including enhanced infiltration or secretion of CD3^+^CD4^+^T cells (6.05±0.12% vs. 3.74±0.73%, *p*<0.05), M1 cells (18.31±1.30% vs. 12.68±0.91%, *p*<0.05), IFNγ (212±20 vs. 87±2 pg/µg protein, *p*<0.001), and IL-12p70 (80±9 vs. 20±2 pg/µg protein, *p*<0.001) and reduced infiltration or expression of M2 cells (7.91±0.89% vs. 13.37±0.95%, *p*<0.05), Treg cells (5.04±0.33% vs. 24.20±4.35%, *p*<0.01), IL-4 (66±11 vs. 100±9 pg/µg protein, *p*<0.05), IL-10 (51±5 vs. 141±19 pg/µg protein, *p*<0.05), and TGF-β1 (0.76±0.13 vs. 1.90±0.23 ng/µg protein, *p*<0.01) compared to PBS administration (Fig. 2A, 2B). However, therapeutic intervention failed to increase the infiltration or expression of CD3^+^CD4^+^ T cells (4.51±0.70%), IFNγ (93±22 pg/µg protein), and IL-12p70 (42±7 pg/µg protein) or attenuate the infiltration or expression of M2 cells (12.23±1.0%), IL-4 (78±12 pg/µg protein), and IL-10 (107±13 pg/µg protein). Therapeutic intervention increased the infiltration of M1 cells (18.16±0.79% vs. 12.68±0.91%, *p*<0.01) and decreased the infiltration or expression of Treg cells (5.86±1.50% vs. 24.20±4.35%, *p*<0.05) and TGF-β1 (0.90±0.11 vs. 1.90±0.23 ng/µg protein, *p*<0.05) in the lung tissue (Fig. 2A, 2B).

To compare the immune response directly regulated by the TLR4/9 agonist complex alone or by tumor cells alone in the lung tissue, the mice injected with B16 cells or PBS were treated with or without the complex for three doses. In the second day after final injection of the complex, the mice were sacrificed and the lung-infiltrating immune cells were analyzed by flow cytometry. The mice treated with the complex without B16 cells increased the infiltration of MHCI^high^ DCs, MHCII^high^ DCs, CD3^+^CD8^+^T cells, and M1 cells and decreased the infiltration of M2 cells and Treg cells in the lung tissues as compared with the PBS-treated control mice (Fig. S2A–F). Compared to the mice treated with the complex with B16 cell inoculation, the mice treated with the complex alone resulted in the increased infiltration of MHCI^high^ DCs, MHCII^high^ DCs, and M1 cells in the lung tissues by 3.5±0.51-, 3.8±0.53-, and 2.0±0.34- fold, respectively (Fig. S2A, S2B, S2D). However, the mice treated with the complex with B16 cell inoculation decreased the infiltration of CD11C^+^MHCI^high^ DCs and CD11C^+^MHCII^high^ DCs, but did not change the infiltration of CTL and M1 cells in the lung tissues as compared with the mice treated with PBS with B16 cell inoculation. In the lung tissues from the mice treated with the complex with B16 cell inoculation, the percentage of M2 cells was increased compared with those from the mice treated with PBS with B16 cell inoculation. These data proved that the application of the TLR4/9 complex without B16 cells activates both innate and adaptive immunity by regulating DC maturation and M1 polarization in the lung. When the TLR4/TLR9 agonist complex is applied after tumor cell inoculation, it is unable to reverse the immunosuppressive tissue environment induced by tumor cells.

Activation of the transcription factors STAT1/STAT3 is crucial in determining whether inflammation in the tumor microenvironment promotes or inhibits cancer development [15], [23]. Because the prophylactic or therapeutic application of the TLR4/TLR9 agonist complex differentially regulated the expression of Th1 cytokines IFNγ and IL-12p70 or Treg cytokine IL-10 (Fig. 2B), which has been coupled with the activation of JAK-STAT1 or STAT3 signaling cascade [23], [24], we examined whether different timing regimens of the TLR4/9 agonist complex differentially regulated the balance of STAT1/3 activity. As shown in Fig. 2C, the phosphorylation or expression of STAT3 and SOCS3 increased, while the phosphorylation or expression of STAT1 and SOCS1 decreased in the lung tissues of the PBS-treated B16-bearing mice as compared to those of PBS-treated control mice. Prophylactic intervention reversed tumor-suppressed phosphorylation or expression of STAT1 (1.82±0.20 vs. 0.46±0.10, *p*<0.001) and SOCS1 and suppressed the tumor-induced phosphorylation or expression of STAT3 (0.72±0.24 vs. 3.74±1.07, *p*<0.05) and SOCS3 in the lung tissues. However, therapeutic intervention could not reverse the tumor cell-induced STAT1 suppression (0.62±0.13) and STAT3 activation (2.65±1.07) in the lung tissues. Perturbation of the STAT1/3 balance induced by the different timing regimens of TLR4/9 agonist complex application directed cytokine/growth factor signals from apoptotic to proliferative or from cancer immunosurveillance to cancer immunoediting.

### Prophylactic, but not therapeutic, application of the TLR4/TLR9 agonist complex activates autophagy in the melanoma cells of metastatic nodes

**Figure 3:**
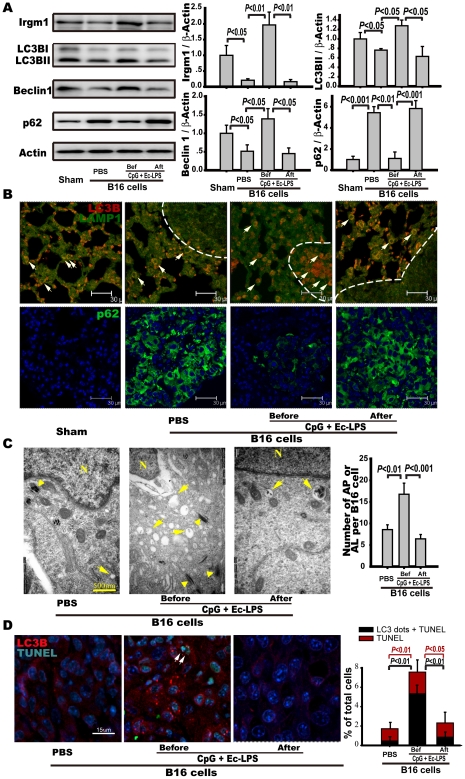
Prophylactic, but not therapeutic, application of TLR4/TLR9 agonist complex activates autophagy in melanoma cells. Mice were treated as indicated in the legend of Figure 1 and sacrificed 14 days after B16 melanoma cell inoculation. The lungs were obtained, and tissue extracts or lung sections were prepared for indicated analysis. (A) The expression of IRGM1, LC3B-II/LC3B-I, beclin-1, and p62 in the lung tissues were detected with Western blot analysis. The representative immune blots are shown in left panel, and statistical results are shown in the right panel. Data are the mean ± S.E. (n = 5). (B) Upper: representative immunofluorescence microphotograph of LC3B and LAMP-1. Lung sections were stained for LC3 (red) and LAMP-1 (green). Arrows point to LC3- and Lamp1- positive cells. Scale bar: 30 µm. Bottom: representative immunofluorescence microphotograph of p62 accumulation. Lung sections were stained for p62 (green) and DAPI (blue). Green areas indicate accumulation of p62 in melanoma cells. Scale bar: 30 µm. (C) Analysis of autophagy in lung sections by transmission electron micrograph (TEM). Autophagosomes and autolysosomes were counted in 10 melanoma cells in every lung section. Data are the mean ± S.E (n = 4 mice/group). Typical autophagosomes and autolysosomes (asterisk) and melanin granules (arrowheads) are indicated. N indicates nuclei of the cell. Scale bar: 500 nm. (D) Co-localization of LC3B immunostaining (red) and TUNEL (green) in the tumor nodes was detected in lung tissue sections. Left three panels are representative images. Arrows point to cells with LC3 dots and TUNEL-positive nuclei. Scale bar: 15 µm. Right panel is bar graph showing the percentage of cells with TUNEL-positive nuclei or with TUNEL positive nuclei and LC3 dots relative to the total number of cells in each section. Twelve images from each lung specimen were counted. Data are presented as the mean ± S.E (n = 6 mice per group).

Autophagy plays many roles as an immunological effector, such as mediating TLR- and Th1 cytokine-induced responses [25]. Previous studies have shown that IRGM1 plays a crucial role in host resistance to a variety of intracellular pathogens by promoting phagolysosome maturation and autophagy. Its expression is induced by the IFNγ/STAT1 signal [26], [27]. We found that the expression levels of IRGM1, LC3B-II, and beclin-1 in the lung of the prophylactically treated B16-bearing mice were markedly increased compared to those in the therapeutically treated and the PBS-treated B16-bearing mice (Fig. 3A). Furthermore, the P62 level was significantly elevated in the lung tissues of therapeutically treated and PBS-treated B16-bearing mice, whereas it was decreased in the lungs of the prophylactically treated B16-bearing mice (Fig. 3A). These data suggest that prophylactic, but not therapeutic, administration of the immune complex activates autophagy in the lungs. To determine where autophagy occurred in the lung sections, autolysosomes or autophagosomes were detected using a confocal microscope and anti-LC3B and anti-LAMP1 antibodies. In the lungs from PBS-treated and therapeutically treated B16-bearing mice, autolysosomes (red and green foci) only occurred at the perimeter of metastasis nodes but not within the nodes (Fig. 3B). However, in the lung tissue from the prophylactically treated mice, autolysosomes were located both at the perimeter and at the center of the nodes (Fig. 3B). Therefore, the number of autolysosomes in metastatic nodes was markedly increased after prophylactic treatment. Meanwhile, what about the changes of autophagic activity in metastatic tumor cells after indicated treatments? p62 is targeted for lysosomal degradation during autophagy, and the expression levels of p62 inversely correlate with autophagic activity [28]. The accumulation of p62 in the lung tissues was examined by confocal microscope. We found that the accumulation of p62 only appeared in metastatic nodes of B16 melanoma cells but not in normal lung tissues, suggesting autophagic activity in melanoma cells is lower than that in normal cells. Furthermore, prophylactic treatment reduced the accumulation of p62 in melanoma cells (Fig. 3B). These data suggest that prophylactic, but not therapeutic, administration of the immune complex activates autophagy in the melanoma cells.

Because we observed that the prophylactic application of the complex promotes cell death (Fig. 1D), we investigated whether cell death depended on complex-activated autophagy [29]. Electron microscopic analysis of melanoma cells in the lung revealed that melanoma cells in the prophylactically treated mice (but not in the therapeutically treated or PBS-treated B16-bearing mice) exhibited a pronounced vacuolization in the cytoplasm and displayed signs of apoptosis (chromatin margination) (Fig. 3C). Consistently, the number of cells with LC3 dots and TUNEL-positive nuclei in the metastatic nodules was markedly enhanced in the prophylactically treated B16-bearing mice (5.3±0.8% vs. 0.5±0.3%, *p*<0.01), but not in the therapeutically treated ones (0.9±0.3% vs. 0.5±0.3%, *p*>0.05) (Fig. 3D). Approximate 70% of TUNEL-positive cells in metastatic nodes were accompanied with LC3 dots in the lung sections from prophylactically treated B16-bearing mice. Moreover, we found that LC3BII and beclin-1 expression and the number of autolysosomes were increased, but cleaved caspase-3 expression was not changed on Day 3 after tumor cell inoculation in the prophylactically treated B16-bearing mice (Fig. S1A-S1C), suggesting that the activation of autophagy preceded apoptosis and that prophylactic administration of the TLR4/9 agonist complex promotes melanoma cell death by stimulating autophagy-associated cell death.

PI3K/Akt/mTOR signaling negatively regulates autophagy [30]. We investigated whether the differential regulation of PI3K/Akt/mTOR signaling was responsible for the different efficacy of two timing regimens against metastasis. PI3K/Akt/mTOR signaling was activated in the lung tissue from PBS-treated B16-bearing mice, as indicated by the enhanced expression or phosphorylation of PI3K (p110α) (1.81±0.23), PI3K (p85α) (3.97±1.0), AKT (2.83±0.47), GSK3 (2.43±0.45), and mTOR (2.29±0.48) (Fig. S3). However, prophylactic intervention caused a significant reduction in the expression or phosphorylation of PI3K (p110α) (0.99±0.20 vs. 2.15±0.65, *p*<0.05), AKT (1.18±0.14 vs. 2.08±0.21, *p*>0.05), GSK3β (1.36±0.12 vs. 4.85 ±1.46, *p*<0.05) and mTOR (1.22±0.21 vs. 2.13±0.50, *p*>0.05) compared to therapeutic intervention (Fig. S3). These results indicate that the prophylactic but not therapeutic administration of the TLR4/9 agonist complex reverses tumor cell-induced activation of the PI3K/AKT/mTOR signaling.

### Neutralization of IFNγ reverses the antimetastatic role of the TLR4/TLR9 agonist complex

**Figure 4:**
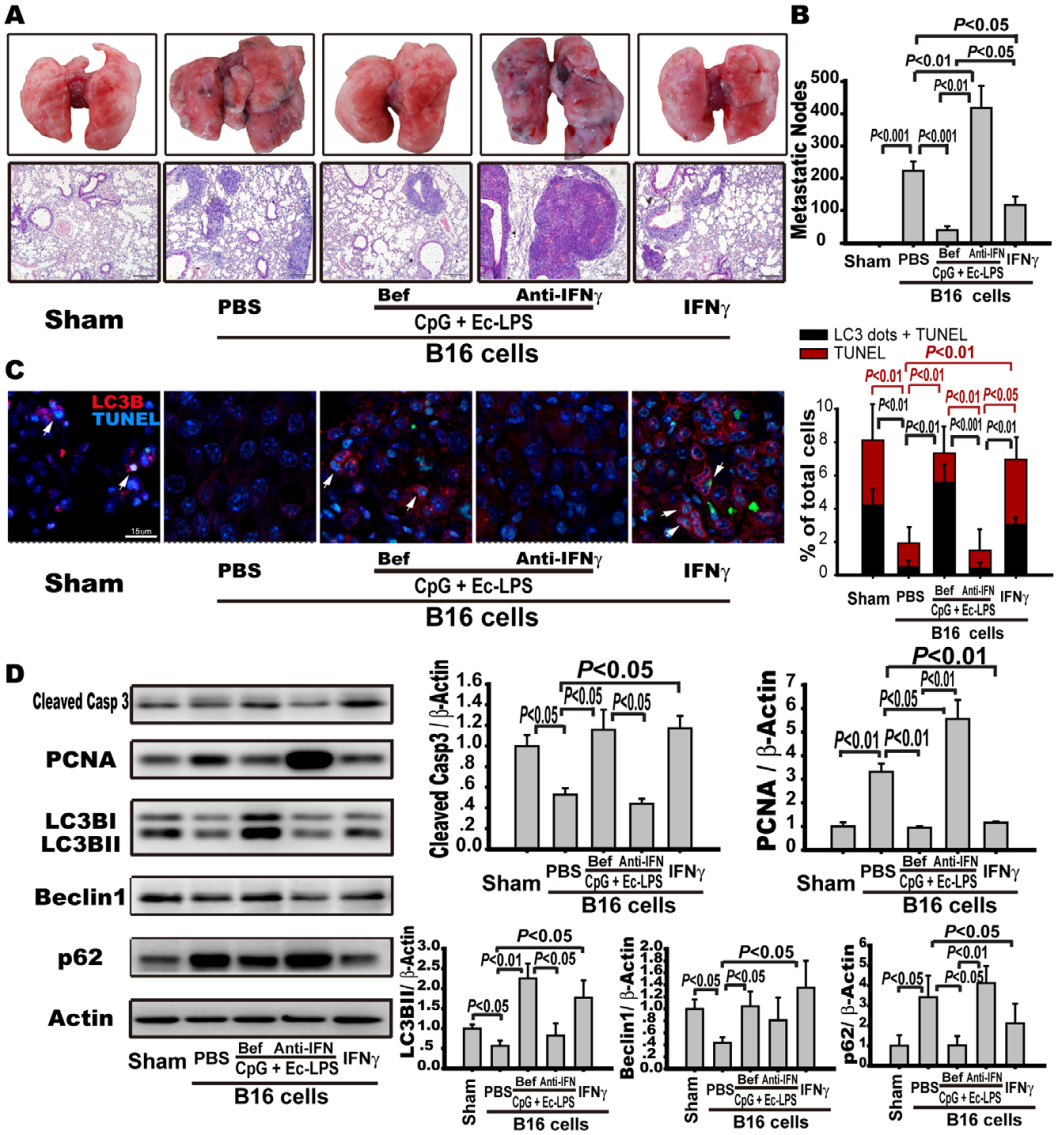
IFNγ neutralization reverses the protective role of TLR4/TLR9 agonist complex against tumor metastasis. C57BL/6 mice were injected with B16-F10 melanoma cells (5×10^5^) and humanely sacrificed 14 days after tumor cell inoculation. The mice were intraperitoneally treated with the TLR4/TLR9 complex (dosage and frequency as in Fig. 1) with or without an IFNγ-neutralizing antibody (100 µg/mouse) or human recombinant IFNγ (1×10^6^ U/kg once a day) before tumor cell inoculation. Control animals were treated with PBS. Externally visible metastases on the lung surface were counted, as described in the legend of Figure 1. (A) Representative lung samples and representative pulmonary H&E staining (magnification: 100×). (B) Metastatic nodules were summarized. Data are the mean ± S.E. (n = 10). (C) Effects of targeting IFNγ on apoptosis or autophagy-associated cell death were evaluated by confocal analysis of LC3 immunostaining (red) and TUNEL (green) in metastatic nodes of lung tissue sections. Left panels are representative immunofluorescence microphotographs of LC3B and TUNEL. Arrows point to cells with LC3 dots and TUNEL-positive nuclei. Scale bar: 15 µm. Right panel is a bar graph to show the percentage of cells with TUNEL-positive nuclei or with TUNEL positive nuclei and LC3 dots relative to the total number of cells in each section. Twelve images from each lung specimen were counted. Data are the mean ± S.E (n = 6 mice/group). (D) The expression of apoptosis and autophagy-related proteins in lung tissues as indicated was detected by Western blots. Representative immune blots are shown in the left panel, and the statistical results are shown in the right panel. Data are presented as the mean ± S.E. (n = 5 mice per group).

To determine whether the activation of IFNγ-STAT1 signaling and autophagy was responsible for the antimetastatic effects produced by the prophylactic administration of the TLR4/9 agonist complex, we examined the antimetastatic role of IFNγ alone and IFNγ-neutralizing antibody plus the TLR4/9 agonist complex treatment. We found that the prophylactic application of IFNγ reduced the number of metastatic nodules by 47±16% and suppressed the phosphorylation or expression of PCNA and P62 while enhancing the phosphorylation or expression of activated caspase-3, LC3BII, beclin-1, and STAT1 as compared to PBS administration in B16-bearing mice (Fig. 4A, 4B, 4D, S4). Consistently, IFNγ treatment enhanced the number of cells with LC3 dots and TUNEL-positive nuclei in metastatic nodes (3.0±0.6% vs. 0.5±0.2%) (Fig. 4C). However, blocking the IFNγ produced by the TLR4/9 agonist complex with an IFNγ-neutralizing antibody almost doubled the number of metastatic nodules compared to PBS administration (425±87 vs. 234±40 nodules/lung, *p*<0.01) (Fig. 4A, 4B). Indeed, blocking IFNγ suppressed apoptosis and autophagy-associated cell death and significantly promoted proliferation, as indicated by the attenuated expression of activated caspase-3, LC3BII, and beclin-1, by decreased the percentage of LC3B positive, LC3B-TUNEL positive, and TUNEL positive cells, and by the enhanced expression of PCNA and accumulation of p62 (Fig. 4C, 4D). Moreover, the prophylactic application of TLR4/TLR9 complex-activated STAT1 was blocked by the IFNγ-neutralizing antibody (Fig. S4). However, therapeutic application of IFNγ or IFNγ plus the complex had no antimetastatic effect on B16-bearing mice (data not shown). These data suggest whether or not the IFNγ/STAT1 signaling and autophagy are activated is critical for the antimetastatic efficacy produced by prophylactic application of the TLR4/TLR9 agonist complex.

### Autophagy activation by rapamycin after tumor inoculation suppresses tumor metastasis

**Figure 5:**
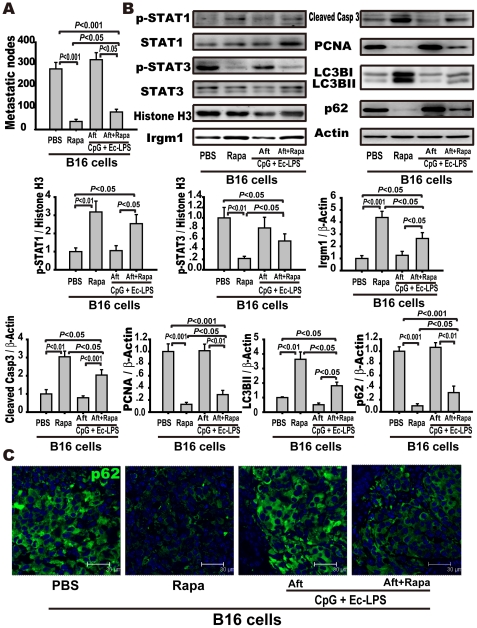
Augmentation of autophagy with rapamycin protects against tumor metastasis. C57BL/6 mice were injected with B16-F10 melanoma cells (5×10^5^) and were humanely sacrificed 14 days after inoculation of tumor cells. Mice were intraperitoneally administered with the TLR4/TLR9 agonist complex (dosage and frequency as in the legend of Fig. 1) with or without rapamycin (10 mg/kg, once a day) after tumor cell inoculation. (A) Rapamycin protects against metastasis. Metastatic nodules were counted and data are presented as the mean ± S.E. (n = 10). (B) Rapamycin activates autophagy and regulates STAT1/3 signaling. The expression of STAT1/3 singling and autophagy-related molecules in the lung tissue was detected by Western blot. The lungs were excised and the cytoplasmic and nuclear fractions were extracted as described in the Methods. The expression of p-STAT1, STAT1, p-STAT3, STAT3, and histone H3 in nucleic extracts and IRGM1, LC3B, cleaved caspase-3, P62, PCNA, and β-actin in the cytoplasm were detected with Western blotting. Left panel is representative western blots and right panels are summary results. Data are presented as the mean ± S.E. of 5 mice per group. (C) Rapamycin treatment decreases p62 accumulation in the lung tissue. Representative immunofluorescence microphotographs were presented to show p62 accumulation in melanoma cells. Lung sections were stained for p62 (green) and DAPI (blue). Scale bar: 30 µm.

To verify that the absence of autophagy activation might be responsible for the complex's failure to elicit an antimetastatic effect after tumor inoculation, rapamycin was administered with or without the TLR4/TLR9 agonist complex after tumor inoculation. Rapamycin is an autophagy activator targeting mTOR. We found that rapamycin, with or without the TLR4/TLR9 agonist complex, markedly decreased the number of tumor metastatic nodes and enhanced the phosphorylation or expression of STAT1, IRGM1, cleaved caspase-3, and LC3BII, while suppressing the phosphorylation or expression of STAT3, PCNA, and P62 compared to PBS (Fig. 5A, 5B). Compared to rapamycin alone, the TLR4/TLR9 agonist complex plus rapamycin did not produce a more potent antimetastatic efficacy (80±12 vs. 37±10 nodules/lung, *p*<0.05) but even partially restrained the antimetastatic activity of rapamycin by suppressing the expression of IRGM1 (2.65±0.48 vs. 4.38±0.51, *p*<0.05) and LC3BII (1.86±0.25 vs. 3.37±0.76, *p*<0.05), and augmenting the phosphorylation of STAT3 (0.56±0.14 vs. 0.22±0.04, *p*<0.05) and the expression of P62 (0.29±0.14 vs. 0.10±0.03, *p*<0.05) in the lung tissues, and by enhancing the accumulation of p62 in metastatic nodes of lung sections (Fig. 5A, 5B, 5C). These data indicate that autophagy is a critical defense mechanism against metastasis independent of immunotherapy.

*Inhibiting STAT3 by AG490 induces anti-tumor activity via activation of STAT1 and autophagy*.

**Figure 6:**
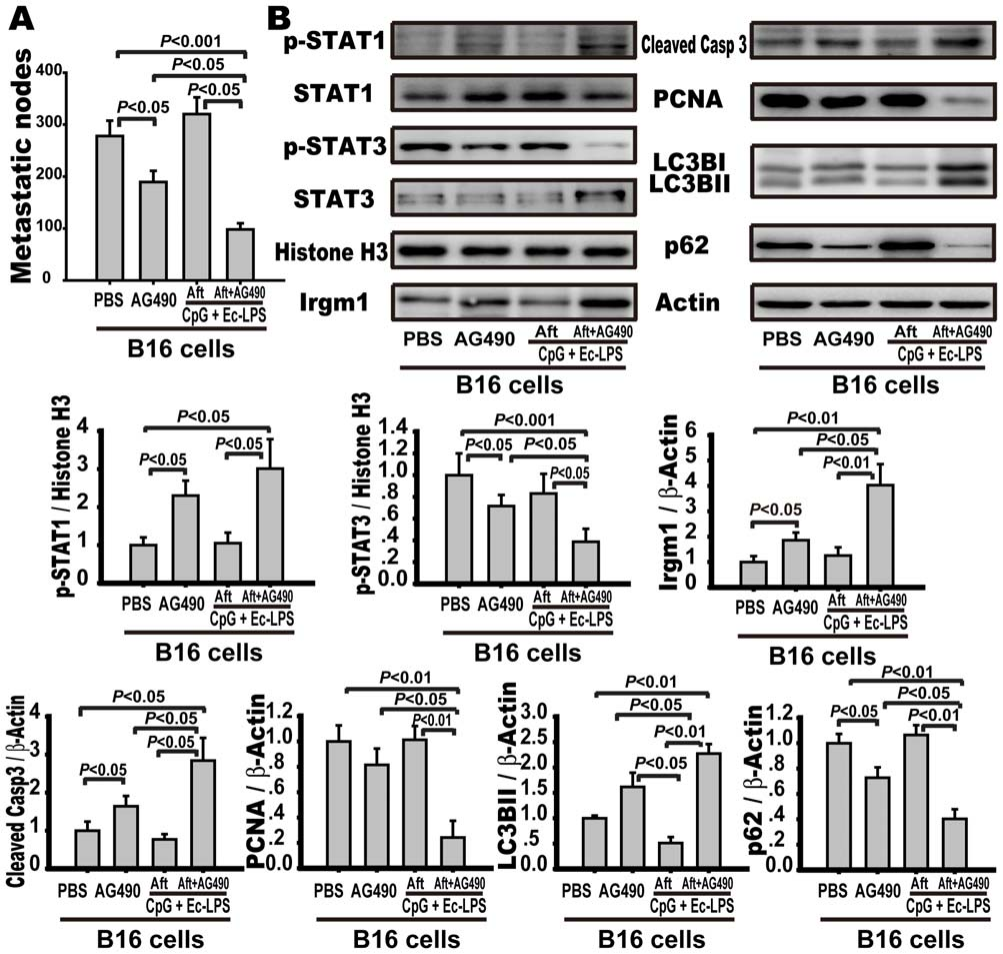
Therapeutic application of TLR4/TLR9 agonist complex and AG490 act synergistically to attenuate metastasis. C57BL/6 mice were injected with B16-F10 melanoma cells (5×10^5^) and were humanely sacrificed 14 days after tumor cell inoculation. The mice were intraperitoneally injected with the TLR4/TLR9 agonist complex (dosage and frequency stated in the legend of Fig. 1) with or without AG490 (30 mg/kg, once a day) after tumor cell inoculation. (A) Metastatic nodules were counted and summarized, and the data are the mean ± S.E. (n = 10). (B) The expression of STAT1/3 singling and autophagy-related molecules in the lung tissue. The lungs were excised and the cytoplasmic and nuclear fractions were extracted as described in the Methods. The expression of p-STAT1, STAT1, p-STAT3, STAT3, and histone H3 in nucleic extracts and IRGM1, LC3B, cleaved caspase-3, P62, PCNA, and β-actin in the cytoplasm were detected with Western blotting. Left panel is representative western blots and right panels are summary results. Data are presented as the mean ± S.E. of 5 mice per group.

**Figure 7:**
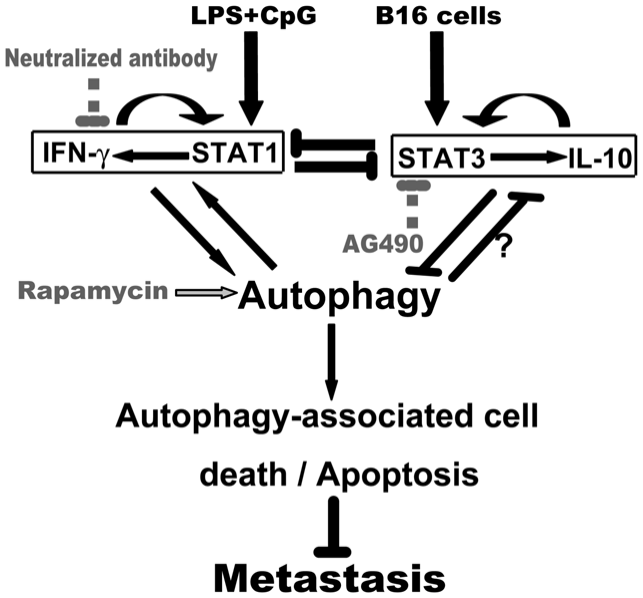
Mechanisms account for the different efficacies of prophylactic or therapeutic TLR4/9 agonist complex against metastasis. Prophylactic application of the TLR4/9 agonist complex results in the activation of IFNγ/STAT1 signaling which stimulates autophagy and autophagy-associated tumor cell death. Neutralization of IFNγ inhibits STAT1 and results in the attenuation of autophagy. Therapeutic application of the TLR4/9 agonist complex can not suppress metastasis because STAT3 is activated and expression of IL-10 is enhanced by tumor cells, which reciprocally results in the suppression of IFNγ/STAT1 signaling, autophagy, and subsequent tumor cell death. Inhibition of STAT3 activation by AG490 (or IL-10 antagonist) or activation of autophagy by rapamycin may suppress metastasis.

Activated STAT3 can suppress STAT1 activity directly or by inducing inhibitory molecules, such as SOCS [20]. To assess whether STAT3 activation restrained the TLR4/TLR9 agonist complex-induced STAT1 activation and autophagy-associated tumor cell death, AG490, a selective JAK/STAT inhibitor, was administered with or without the complex after tumor inoculation. Mice treated with AG490 alone showed an antimetastatic effect with decreased lung metastatic nodes (189±22 vs. 278±29 nodules/lung, *p*<0.05), STAT3 suppression, STAT1 activation and IRGM1 expression when compared to the PBS-treated B16-bearing mice (Fig. 6A, 6B). However, the administration of the TLR4/TLR9 complex plus AG490 resulted in a further reduction of metastatic nodules (98±12 vs. 189±22 nodules/lung, *p*<0.05) with the activation of caspase-3 (2.84±0.6 vs. 1.42±0.27, *p*<0.05) and autophagy in the lungs (Fig. 6A, 6B). Additionally, the mice treated with the TLR4/TLR9 agonist complex plus AG490 showed a higher level of STAT3 suppression and IRGM1 expression compared to the mice treated with or without the TLR4/TLR9 complex (Fig. 6B). These data indicate that the inhibition of STAT3 reverses the suppressed STAT1 activity and autophagy caused by tumor cells, which produces anti-metastatic efficacy (Fig. 7).

## Discussion

Despite significant advances in cancer immunology and immunotherapy, clinical investigations have had marginal success [6], [31]. The reasons underlying the relatively low clinical responses to immunotherapy in cancer patients include 1) suboptimal synergistic combinations of immunotherapeutic agents and 2) delayed timing for administering the immunotherapeutic agents. Regarding the first reason, recent studies indicate that there is not only insufficient antitumor immunity, but also too many immunosuppressive factors existing in the tumor environment [6], [32]. Thus, the optimal synergistic combinations of immunotherapy should include components that can enhance the antitumor capability and components that can eliminate the tumor-promoting factors from the tumor environment [19]. Regarding the second reason, immunotherapy should be applied as early as possible, rather than at a later stage of the disease or after other treatments have failed in the clinical trial. For instance, beginning immunotherapy a day or two before surgery can boost the immune system and block its suppression by psychological and physiological stress [33].

In current study, we assessed the efficacy of an immunotherapeutic regimen consisting of the TLR4 agonist EC-LPS plus the TLR9 agonist CpG ODN against tumor metastasis. TLR agonists have been shown to be Myd88-associated TLR (TLR2, TLR5, TLR7 and TLR9) agonists and TRIF-coupled TLR (TLR3 and TLR4) agonists that can act in synergy to induce high levels of proinflammatory cytokines when applied simultaneously [14]. Furthermore, TLR agonists acting in synergy showed an increased and sustained capacity to prime Th1 responses [14], [34]. It has been established that Th1 responses are crucial for protection against tumor development and progression. Our data show that triggering TLR4 and TLR9 simultaneously with LPS plus CpG before tumor inoculation inhibits tumor metastasis significantly, whereas triggering either TLR4 or TLR9 has no effect on metastasis (data not shown). However, the potent immunotherapeutic complex can only prevent disease and is unable to therapeutically suppress metastasis, similar to the failures of immunotherapy seen in patients with late-stage cancer [10], [35], suggesting that timing is crucial for efficacious anticancer immunotherapy.

We found that the prophylactic or therapeutic application of the TLR4/TLR9 agonist complex differentially regulated Th1 responses and subsequent tumor cell death by activating IFNγ/STAT1 signaling (in the case of prophylactic treatment) or by activating STAT3 (in the case of therapeutic treatment), which is responsible for the different efficacy against tumor metastasis. These findings are consistent with reports that STAT1 and STAT3 play opposite roles in cancer immunity [23] and that IFNγ/STAT1 activation is important in TLR agonist-induced cellular inflammation [14]. Although the precise mechanism is required further investigation, tumor cell-induced STAT3 activation may largely be responsible for the suppression of IFNγ/STAT1 signaling and Th1 responses in mice treated with the TLR4/9 agonist complex after tumor cell inoculation. We and others have previously shown that the constitutive activation of STAT3 in melanoma cells determines the development of tumor immune tolerance and tumor progression [15], [36]. Furthermore, STAT3 can be induced directly and rapidly by TLR4 and TLR9 agonists [37]. For the reciprocal regulation of STAT1/3 activity, STAT3 inhibition by JAK/STAT antagonist AG490 may allow STAT1 activation and the expression of antitumor cytokines to suppress tumor metastasis. In fact, therapeutic administration of the TLR4/9 agonist complex plus AG490 is able to suppress the STAT3 activity, and the anti-metastatic efficacy is thus augmented or restored compared to the AG490 or TLR4/9 complex treatment alone (Fig. 7).

The role of autophagy in tumorigenesis and metastasis remains controversial because autophagy either promotes cell death or cell survival [38], [39]. However, the induction of autophagy- associated cell death has been identified as a crucial tumor-suppressing mechanism. Our results now clearly demonstrate that the autophagy-associated cell death is involved in the mechanism by which the prophylactic application of the TLR4/9 agonist complex promotes B16 melanoma cell apoptosis. Notably, the autophagy process is commonly regulated by the cytokines and transcription factors in tumor microenvironment or by the tumor itself [29]. Our studies indicate that autophagy activation in tumor cells from the mice treated prophylactically with the TLR4/9 agonist complex is associated with the elevated levels of IFNγ expression and STAT1 phosphorylation. In contrast, IFNγ/STAT1 signaling and autophagy are not activated in tumor cells from the lungs of therapeutically treated mice. Indeed, IFNγ neutralization alone suppressed STAT1 activation and autophagy in the lung tissues from the prophylactically treated mice, which resulted in a deprivation of the TLR4/9 agonist complex-induced antimetastatic effect. Through reversing the activated STAT3 by AG490, the suppressed STAT1 activity and autophagic activity were restored, which led to an antimetastatic effect in mice treated therapeutically with the TLR4/9 complex. Furthermore, rapamycin, which induces autophagy by inhibiting mTOR kinase, enhances STAT1 activity in the lungs of B16-bearing mice and produces a potent anti-metastatic action. These data suggest that IFNγ/STAT1-activated autophagy is critical for the anti-metastatic role of the TLR4/9 agonist complex. Consistent with our findings, Li et al found that suppressing STAT1 phosphorylation by fludarabine or by silencing the expression of STAT1 inhibits the expression of LC3BI/II and decreases the number of autophagosomes induced by IFN-γ in primary human macrophages [40]. However, Chang et al. reported that embryonic fibroblasts from autophagy-deficient mice are resistant to IFNγ-induced STAT1 activation [24]. Thus, STAT1 can interact positively with autophagy although price mechanism requires to be identified (Fig. 7).

On the other hand, our studies indicate that therapeutic treatment of mice with the TLR4/9 agonist complex after inoculation of B16F10 melanoma cells can not reverses tumor cell-induced STAT3 activation, IL-10 expression, and autophagy suppression in the lung tissues. Similarly to the IFNγ/STAT1 signaling, STAT3 and IL-10 can form a positively regulatory loop to promote tumor progression and metastasis through sustaining immunosuppressive environment in tumor tissue [41]. Van Grol et al recently reported that IL-10 suppressed autophagy induced by rapamycin via activation of STAT3 partially [41] while Park et al report that IL-10 inhibits autophagy in macrophages via activation of PI3K pathway[42]. Thus, activation of IL-10/STAT3 can impair autophagy induction and decrease autophagy-associated tumor cell death. Interestingly, we observe that direct activation of autophagy causes a significant inhibition of STAT3 activity although we do not know what mechanism responsible for this regulation at this time. These studies indicate that IFNγ/STAT1 signaling plays a critical role in autophagy induction by TLR agonists, and IL-10/STAT3 signaling serves as a negative modulator of autophagy in response to tumor cells (Fig. 7).

In addition to the balance of STAT1/3 signaling, PI3K signaling may be an alternative mechanism responsible for the different antimetastatic roles produced by preventive or therapeutic administration of the TLR4/9 agonist complex. Autophagy acts as an immunological effector for TLR activation and Th1 cytokines [43]. However, the PI3K pathway functions as a negative regulator of TLR responses to downregulate autophagy in cancer cells [44]. The present study indicates that prophylactic application of the TLR4/9 agonist complex decreases the expression of PI3K p85 and p110 subunits, suppresses the activation of AKT and the AKT downstream target GSK-3β, which results in mTOR inactivation and autophagy activation. However, the activation of PI3K/AKT/mTOR signaling by B16-F10 cells cannot be suppressed with a therapeutic application of the TLR4/9 agonist complex, which even further augments the activated PI3K-AKT-mTOR signaling and reverses partially the rapamycin-induced attenuation of mTOR [41]. These findings indicate that the differentiated regulation of PI3K/AKT/mTOR signaling is also responsible for the different efficacies caused by prophylactic or therapeutic administration of the TLR4/9 agonist complex.

In summary, we have identified a mechanism underlying the failure of an immunotherapeutic protocol against tumor progression and metastasis; the tumor cells activate STAT3 to hijack host immune cells to protect the IFNγ/STAT1 signaling from activation and subsequently protect tumor cells from autophagy-associated cell death (Fig. 7). Moreover, we demonstrate that autophagy is a suppressive mechanism of metastasis and is regulated by the tumor microenvironment. Our studies not only suggest that administration timing is crucial for an efficacious cancer immunotherapy but also indicate a novel strategy to induce an effective antimetastatic response. Immunotherapy plus an anti-inflammatory agent (AG490) or autophagy activator (rapamycin) may be a rational immunotherapy against tumor progression and metastasis.

## Supporting Information

Figure S1 **Therapeutic application of the TLR4/9 agonist complex suppressed apoptosis and autophagy in the metastatic cells.** The mice were sacrificed on the 3^rd^ day after B16 melanoma cell inoculation. Lung tissue extracts were prepared as described in the Methods. (A) Western blot analysis and the corresponding quantification of cleaved caspase-3 and PCNA in lung tissues 3 days after tumor cell inoculation. Data are mean ± S.E. (n = 5). (B) Western blot analysis and the corresponding quantification of the autophagy-related proteins LC3B-II/LC3B-I, beclin-1 and p62 in lung tissues. Data are mean ± S.E. (n = 5). (C) Representative immunofluorescence microphotograph of LC3 and LAMP-1. Lung tissue sections were stained for LC3 (red) and LAMP-1 (green). Arrows point to LC3- and Lamp1- positive cells. Scale bar: 30 µm.
(TIF)

Figure S2 **Therapeutic administration of the complex fails to reverse the tumor cells-induced suppressive immune responses.** Mice were i.v. injected with B16 melanoma cells (5×10^5^/mouse) (10 mice/group) or with equal volume of PBS (10 mice/group). Five of B16-bearing mice and five of PBS-treated mice were administered with the TLR4/9 agonist complex for three doses as indicated in the legend of Figure 1. The mice were sacrificed on the second day after the final dose of the complex administration. The lung single-cell suspensions were prepared as indicated in the Methods. CD11c^+^MHC I^high^ cells (A), CD11c^+^MHC II^high^ cells (B), CD3^+^CD8^+^ T cells (C), CD11b^+^F4/80^+^CD206^−^ M1 macrophages (D), CD11b^+^F4/80^+^CD206^+^ M2 macrophages (E) and Foxp3^+^CD4^+^CD25^+^ Treg cells (F) were detected with flow cytometry. The data are represented as the mean percentage of positive cells ± S.E. (n = 6).
(TIF)

Figure S3 **Prophylactic or therapeutic application of the TLR4/9 agonist complex differentially regulated PI3K-AKT-mTOR signaling.** Mice were treated as indicated in the legend of Figure 1 and sacrificed on the 14th day after B16 melanoma cell inoculation. Lung tissue extracts were prepared as described in the Methods. Lung tissue extracts were prepared as described in the Methods. The expression of PI3K110α, PI3K85α, p-AKT, AKT, p-GSK3, GSK3, p-mTOR and mTOR was detected by western blot. Data are mean ± S.E. (n = 5).
(TIF)

Figure S4 **Neutralization of IFNγ regulated STAT transcriptional activity.** Mice were treated as indicated in the legend of Figure 1 and sacrificed on the 14th day after B16 melanoma cell inoculation. Lung tissue extracts were prepared as described in the Methods. The expression of p-STAT1, STAT1, p-STAT3, STAT3 and histone H3 in the nucleus and SOCS1, SOCS3 and β-actin in the cytoplasm were detected by western blot. The left panel represents immune blots, and the right panel is the related statistical results. Data are mean ± S.E. (n = 5).
(TIF)
