# Supplementary figures and images for: Timing Is Critical for an Effective Anti-Metastatic Immunotherapy: The Decisive Role of IFNγ/STAT1-Mediated Activation of Autophagy
Source: PLoS One. 2011 Sep 13;6(9):e24705. doi: 10.1371/journal.pone.0024705 (PMC3172290; doi:10.1371/journal.pone.0024705)

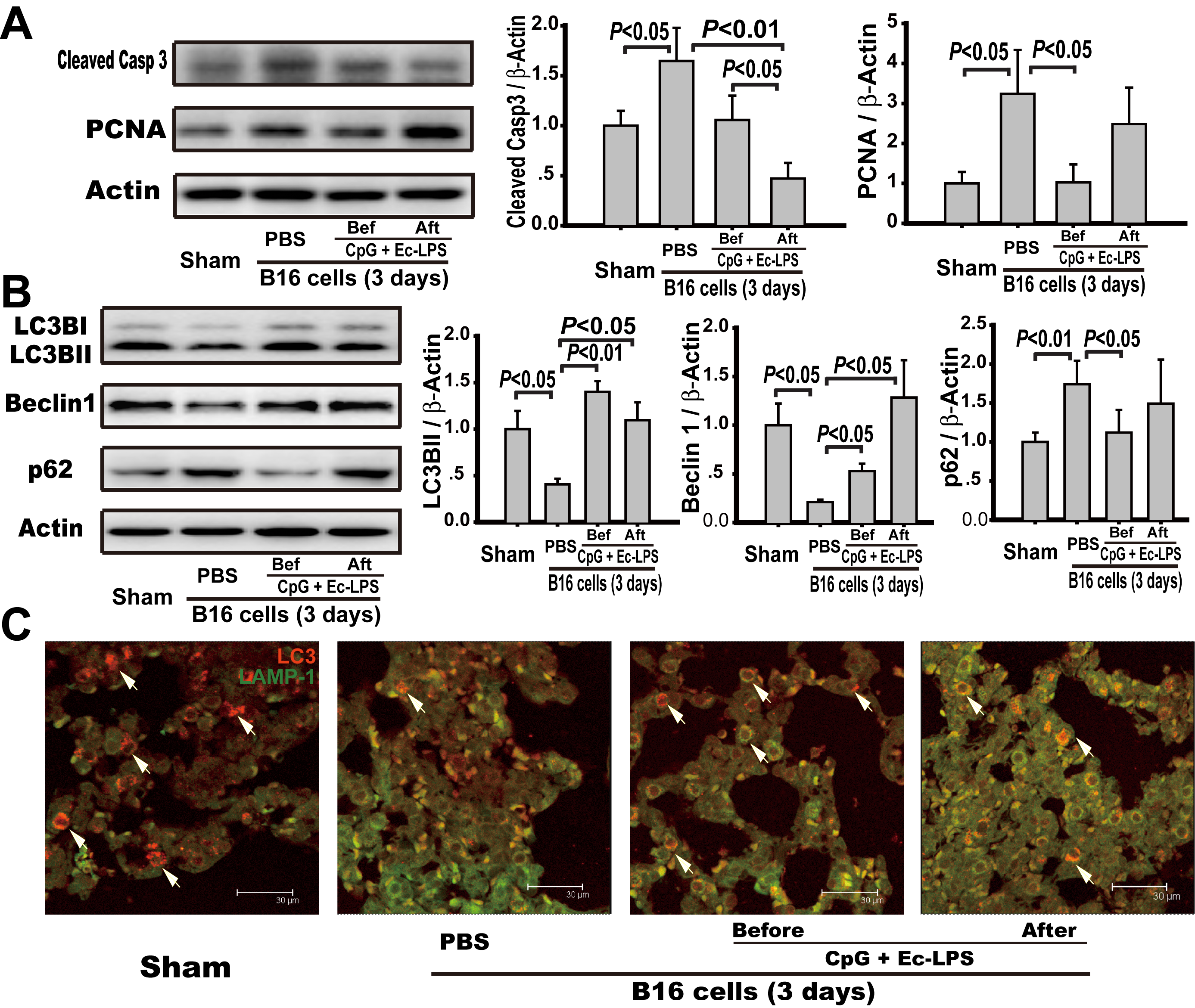

Supplement: Figure S1 — Therapeutic application of the TLR4/9 agonist complex suppressed apoptosis and autophagy in the metastatic cells. The mice were sacrificed on the 3rd day after B16 melanoma cell inoculation. Lung tissue extracts were prepared as described in the Methods. (A) Western blot analysis and the corresponding quantification of cleaved caspase-3 and PCNA in lung tissues 3 days after tumor cell inoculation. Data are mean ± S.E. (n = 5). (B) Western blot analysis and the corresponding quantification of the autophagy-related proteins LC3B-II/LC3B-I, beclin-1 and p62 in lung tissues. Data are mean ± S.E. (n = 5). (C) Representative immunofluorescence microphotograph of LC3 and LAMP-1. Lung tissue sections were stained for LC3 (red) and LAMP-1 (green). Arrows point to LC3- and Lamp1- positive cells. Scale bar: 30 µm. (TIF) [file pone.0024705.s001.tif]

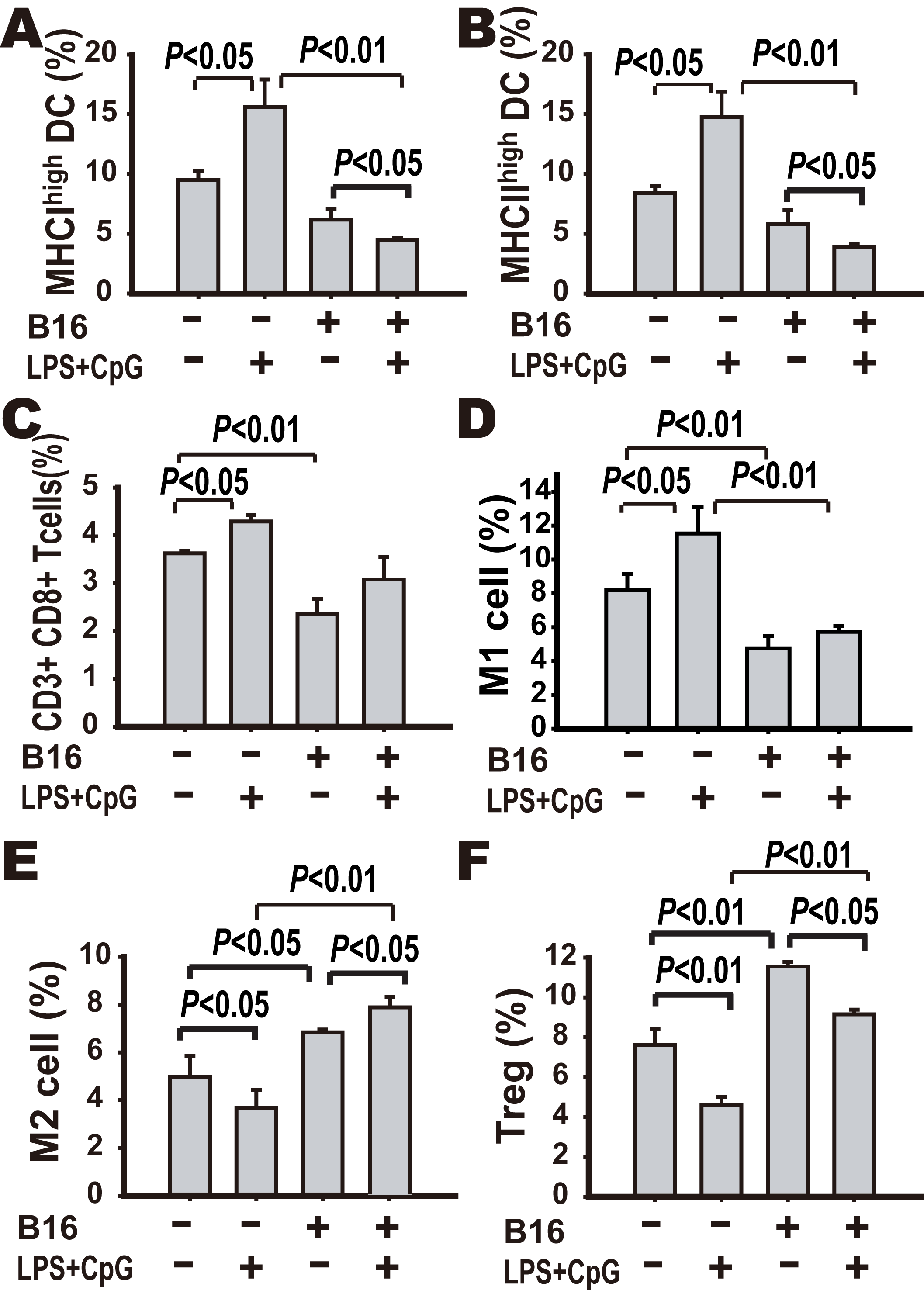

Supplement: Figure S2 — Therapeutic administration of the complex fails to reverse the tumor cells-induced suppressive immune responses. Mice were i.v. injected with B16 melanoma cells (5×10^5^/mouse) (10 mice/group) or with equal volume of PBS (10 mice/group). Five of B16-bearing mice and five of PBS-treated mice were administered with the TLR4/9 agonist complex for three doses as indicated in the legend of Figure 1. The mice were sacrificed on the second day after the final dose of the complex administration. The lung single-cell suspensions were prepared as indicated in the Methods. CD11c+MHC Ihigh cells (A), CD11c+MHC IIhigh cells (B), CD3+CD8+ T cells (C), CD11b+F4/80+CD206− M1 macrophages (D), CD11b+F4/80+CD206+ M2 macrophages (E) and Foxp3+CD4+CD25+ Treg cells (F) were detected with flow cytometry. The data are represented as the mean percentage of positive cells ± S.E. (n = 6). (TIF) [file pone.0024705.s002.tif]

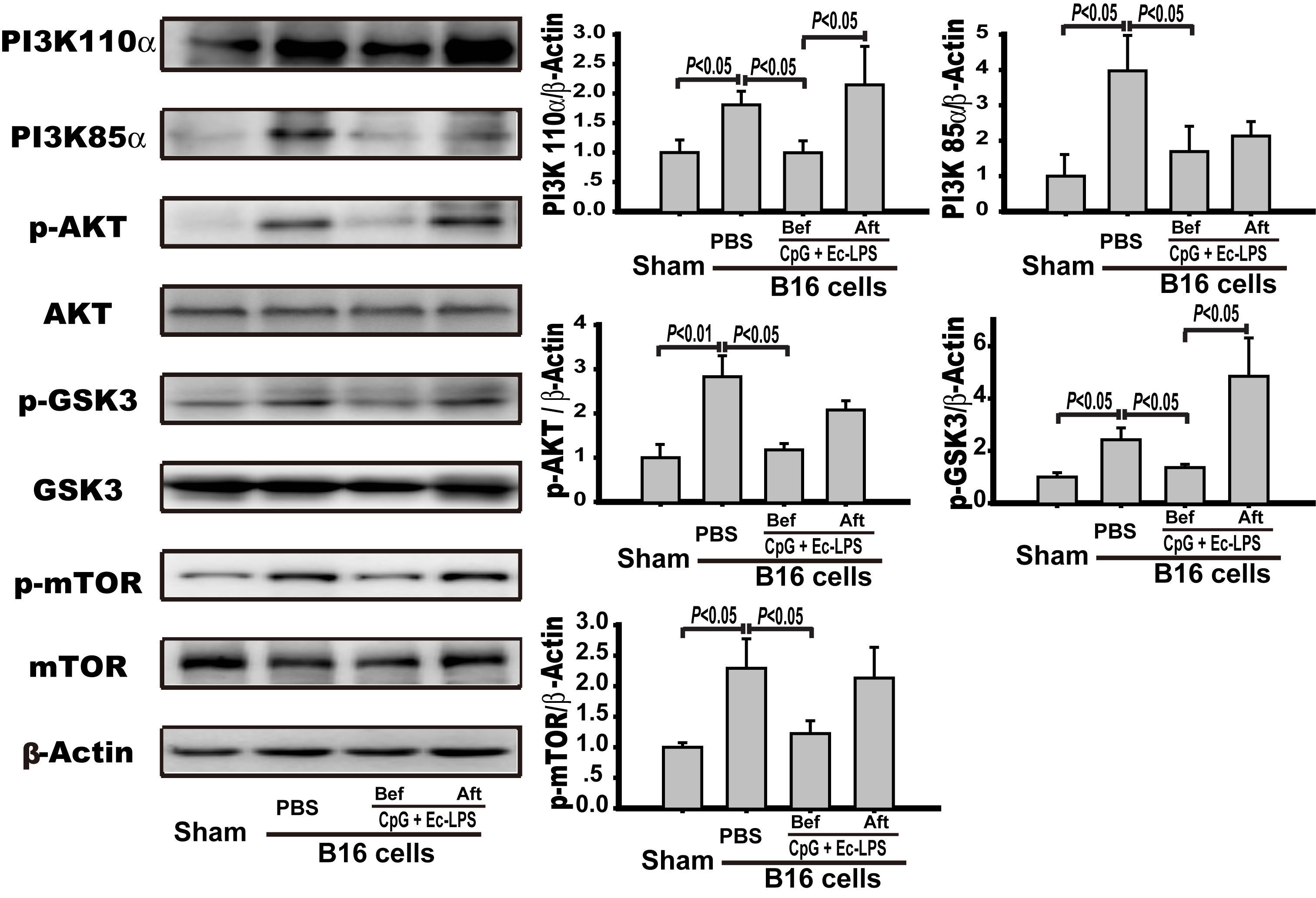

Supplement: Figure S3 — Prophylactic or therapeutic application of the TLR4/9 agonist complex differentially regulated PI3K-AKT-mTOR signaling. Mice were treated as indicated in the legend of Figure 1 and sacrificed on the 14th day after B16 melanoma cell inoculation. Lung tissue extracts were prepared as described in the Methods. Lung tissue extracts were prepared as described in the Methods. The expression of PI3K110α, PI3K85α, p-AKT, AKT, p-GSK3, GSK3, p-mTOR and mTOR was detected by western blot. Data are mean ± S.E. (n = 5). (TIF) [file pone.0024705.s003.tif]

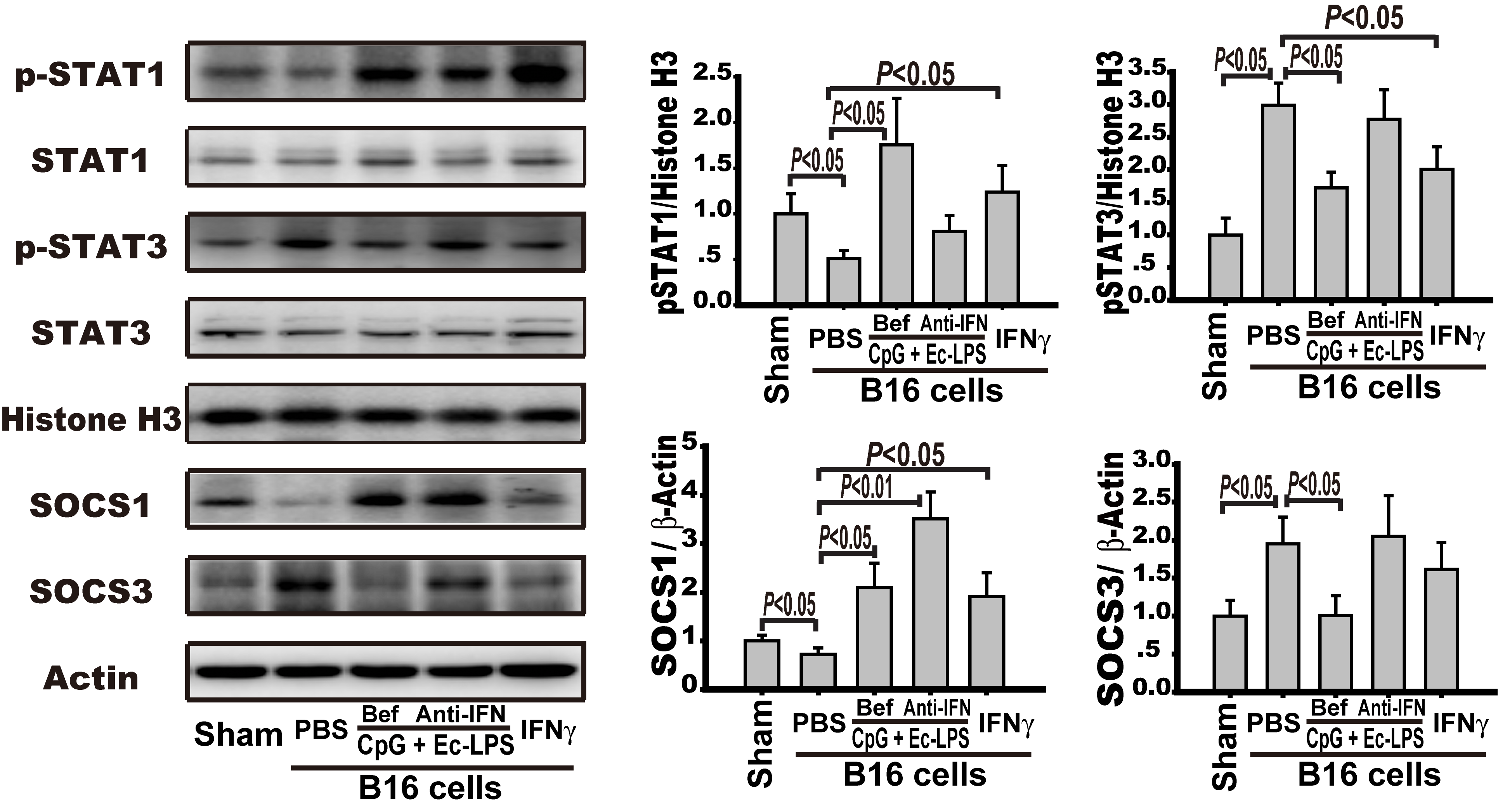

Supplement: Figure S4 — Neutralization of IFNγ regulated STAT transcriptional activity. Mice were treated as indicated in the legend of Figure 1 and sacrificed on the 14th day after B16 melanoma cell inoculation. Lung tissue extracts were prepared as described in the Methods. The expression of p-STAT1, STAT1, p-STAT3, STAT3 and histone H3 in the nucleus and SOCS1, SOCS3 and β-actin in the cytoplasm were detected by western blot. The left panel represents immune blots, and the right panel is the related statistical results. Data are mean ± S.E. (n = 5). (TIF) [file pone.0024705.s004.tif]
